# Assessment of personality-related levels of functioning: a pilot study of clinical assessment of the DSM-5 level of personality functioning based on a semi-structured interview

**DOI:** 10.1186/s12888-016-1011-6

**Published:** 2016-08-25

**Authors:** Birgitte Thylstrup, Sebastian Simonsen, Caroline Nemery, Erik Simonsen, Jane Fjernestad Noll, Mikkel Wanting Myatt, Morten Hesse

**Affiliations:** 1 Center for Alcohol and Drug Research, Aarhus University, Bartholins Allé 10, 8000 Aarhus C, Denmark; 2 Regional Services for Mental Health, Capital Region, Stolpegårdsvej 20, 2820 Gentofte, Denmark; 3 BOMI, Renter for Neurorehabilitation, Maglegårdsvej 15, Roskilde, Denmark; 4 Regional Services for Mental Health, Nørregade 54, 4100 Ringsted, Denmark

**Keywords:** Personality, Levels of functioning, Assessment, Interview, Clinical practice

## Abstract

**Background:**

The personality disorder categories in the Diagnostic and Statistical Manual of Mental Disorders IV have been extensively criticized, and there is a growing consensus that personality pathology should be represented dimensionally rather than categorically. The aim of this pilot study was to test the Clinical Assessment of the Level of Personality Functioning Scale, a semi-structured clinical interview, designed to assess the Level of Personality Functioning Scale of the DSM-5 (Section III) by applying strategies similar to what characterizes assessments in clinical practice.

**Methods:**

The inter-rater reliability of the assessment of the four domains and the total impairment in the Level of Personality Functioning Scale were measured in a patient sample that varied in terms of severity and type of pathology. Ratings were done independently by the interviewer and two experts who watched a videotaped Clinical Assessment of the Level of Personality Functioning Scale interview.

**Results:**

Inter-rater reliability coefficients varied between domains and were not sufficient for clinical practice, but may support the use of the interview to assess the dimensions of personality functioning for research purposes.

**Conclusions:**

While designed to measure the Level of Personality Functioning Scale with a high degree of similarity to clinical practice, the Clinical Assessment of the Level of Personality Functioning Scale had weak reliabilities and a rating based on a single interview should not be considered a stand-alone assessment of areas of functioning for a given patient.

## Background

Perhaps nothing is more central to treating mental health problems than the patient’s personality [1]. Since the introduction of Axis II into the diagnostic nomenclature in the DSM-III, it has been possible for clinicians and researchers to consider personality in practice and research. However, the personality disorder (PD) categories in the Diagnostic and Statistical Manual of Mental Disorders IV [2] have been extensively criticized on a number of grounds. For instance, it has been noted that there is considerable overlap between categories in both general population [3] and clinical samples [4], that most of the PD diagnoses do not represent categorical phenomena at the latent variable level [5], and that the way in which clinicians diagnose PD does not correspond to the way that researchers have found to be the most reliable and valid [6].

There is now a growing consensus that personality pathology should be represented dimensionally rather than categorically [5, 7, 8]. While the DSM-IV categorical model was retained in the DSM-5 Section II as the official diagnostic system, a novel approach to the assessment of personality pathology was included in Section III to stimulate further research and possible inclusion in future DSM iterations [9]. The new system is a hybrid of dimensional and categorical ratings that include personality traits as well as diagnoses [10]. An innovative component is the Level of Personality Functioning Scale (LPFS), which defines personality pathology in terms of impairments in self-functioning (Identity and Self-direction) and interpersonal functioning (Empathy and Intimacy), and can be used to assess both the presence and severity of personality pathology [9]. The four domains are rated individually, and for diagnostic purposes the clinician selects the level of functioning that most closely captures the patient’s overall level of impairment [11]. The LPFS constitutes the first step toward the diagnosis of a personality disorder under Section III [10]. Following the LPFS assessment, the clinician must assess pathological personality traits according to five trait domains: negative affectivity, detachment, antagonism, disinhibition, and psychoticism.

### The diagnostic assessment of the level of personality functioning scale

During the development of the LPFS, Morey and colleagues first published data in support of the validity of a global dimension of personality pathology related to both self and interpersonal functioning [11], and in a subsequent study, Morey and colleagues found support for the concurrent and clinical validity of the LPFS [12]. Similarly, other measures such as the Inventory of Personality Organization and the Objects Relations Inventory have been used as indicators of levels of personality functioning [13]. A study by Hopwood and colleagues [14] of PD patients participating in the Collaborative Longitudinal Personality Disorders Study [15] demonstrated that generalized severity is the most important single predictor of concurrent and prospective dysfunction in the assessment of personality pathology, and may be one of the most important features to assess when working with personality pathology [11, 16].

The alternative model has received substantial criticism after its publication, especially for being too complicated for general clinical use and research [10]. While this criticism may in part reflect the difficulty of adjusting to new ideas when conducting any assessment or intervention, it underlines the importance of finding the right balance between the time and resources used when obtaining qualified assessment tools for clinical use as well as research. However, there is at present no officially approved clinical instrument to assess the LPFS, and since a substantial amount of evidence from clinical research points to the difficulties in obtaining valid and reliable diagnostic information about personality pathology, there is a need for a diagnostic instrument that can be used to assess each particular aspect of the LPFS in a standardized, reliable, meaningful and clinically acceptable way. Several research groups are currently working on developing a standardized instrument, but to the best of our knowledge, only two studies have published data on the inter-rater reliability of the LPFS ratings.

To challenge the claim that the constructs in the LPFS are too complex for most clinicians to rate e.g. [1, 17–19], Zimmerman and colleagues [20] studied whether 22 psychology undergraduate students were able to apply the LPFS with sufficient reliability. The ratings were based on an operationalized psychodynamic interview of 10 female patients conducted by an experienced clinician, of which five patients were diagnosed with a PD according to the SCID-II and five were not. The study indicated that the concerns about the complexity of the LPFS constructs were premature with an acceptable Intra Class Correlation (ICC) for the total dimension (ICC) = .51, 95 % confidence intervals [CI] (.31, .78). However, the ICC was more mixed for the four domains, ranging from .25 for Empathy to .63 for Intimacy. Although a social relations model analysis found evidence of significant perceiver variance for Empathy, the students’ ratings converged with expert-rated proxy measures of the severity of personality pathology, that is the presence and number of patients with DSM-IVPD diagnoses and OPD level of structural integration. The second reliability study was conducted by Few and colleagues [21] with 109 community adults receiving outpatient mental health treatment. In this study, the LPFS was rated by trained graduate students based on a video-taped SCID-II interview conducted by graduate students, and the reliability was based on both interviewer and video-ratings. In this study, the inter-rater reliabilities for Identity, Self-Direction, Empathy, and Intimacy were .49, .47, .49 and .47, respectively.

These two studies have contributed significantly to the ongoing research regarding the LPFS, although there are reasons for concern about the clinical generalizability of the inter-rater reliability in both. For one, both studies involved patients diagnosed with a PD at the low to moderate end of the severity continuum, which corresponds to levels zero to two in the LPFS, and did not involve patients with a more severe PD, such as schizotypal, paranoid and antisocial PD, corresponding to levels three and four in the LPFS. Secondly, the LPFS was assessed by untrained and inexperienced raters in the Zimmerman study, and the findings might therefore set a lower bound for the inter-rater reliability. Also, in both studies, the ratings were based on interviews which are hardly representative of a standard clinical interview. In the Few study, both the interviews and ratings were carried out by specifically trained graduate students, and also in this context it seems unlikely that a general clinician would conduct and rate the LPFS in a similar way.

Finally, Hutsebaut and colleagues developed a semi-structured interview to assess the LPFS [22], the Alternative Model for Personality Disorders (AMPD). Each section of the AMPD opens with a general question, but specific questions are asked that probe directly for facets of the LPFS. The inter-rater reliability of the AMPD is substantially higher than the inter-rater reliability that has been reported in the two previous studies, with intraclass correlations ranging from .58 to .82 in the clinical sample, and from .81 to .92 when combining the patient sample with additional non-clinical cases.

### The Clinical Assessment of the Level of Personality Functioning Scale (CALF)

The aim of the present study was to assess inter-rater reliability of a semi-structured interview developed for the assessment of the four domains in the LPFS, the Clinical Assessment of the Levels of Personality Functioning Scale (CALF). The CALF was designed to be relatively brief, lasting less than an hour, and to be suitable early on in the assessment process. In order to assess duration of interview and identify questions that were problematic, or areas that required further questions before the current study, a previous version of the CALF was tested with patients undergoing treatment for substance use disorders and prison inmates in a high-security prison for offenders who were deemed to need treatment for severe psychiatric disorders, and a small community sample.

Further we wanted to increase the diversity of personality pathology studied with the LPFS by sampling a range of patients who varied in terms of both severity and type of pathology within different treatment settings. If the inter-rater reliability was also acceptable under such conditions, this would increase the acceptability of using the CALF to assess patients based on the LPFS in clinical practice, making a strong case against the need for the costly retraining of expert clinicians to carry out the clinical interviews and rate the LPFS.

## Methods

### Procedure

To have access to a range of patients who varied in severity and type of pathology in this study, we sampled patients from three different sources: an outpatient psychotherapy clinic that specifically served patients with PDs, a general outpatient psychiatric clinic, and substance dependent patients in ongoing day or residential treatment with no drug or alcohol use in the past 30 days, including some from a high security prison. All participants were informed about the aim and content of the study and gave informed consent to participate.

The CALF interviews were conducted by six trained experts and lasted for between 44 and 69 min (M = 57.3, SD = 9.6); all interviews were videotaped. Following this, the videotaped interviews were distributed between six experts with each interview co-rated by two experts, who had not carried out the interview that was being rated.

### Interviewers and raters

The interviews and ratings were conducted by four psychologists and two MDs with extensive experience with clinical assessment and assessment research, three men and three women with a mean age of 38.9 years (range 31 to 46 years). Additionally, two of the interviews were conducted by a female psychology student, age 31 years, under supervision by one of the MDs.

### Participants

Participants were recruited from a patient (*n* = 36) and a community sample (*n* = 7). The patient sample consisted of 36 patients, 19 men and 17 women, with a mean age of 36 years (range: 18 to 56 years): Substance dependent patients (*n* = 19); Personality disordered patients (*n* = 12); Patients with anxiety or depression (*n* = 5). The community sample consisted of seven women with a mean age of 34 years (range: 24 to 45 years).

#### Measures

*The Level of Personality Functioning Scale* (LPFS) rates the four domains Identity and Self-direction (self-functioning) and Empathy and Intimacy (interpersonal functioning) on a scale from 0 (no impairment) to 4 (highest level of impairment). Within each domain, a comprehensive description is given for each criterion. For the purpose of this study, the total score was summarized as the mean of the four domain scores.

No formal training was provided for the raters, and the instructions for determining the ratings were restricted to handing out written copies of the LPFS.

#### The CALF interview

Like the Metacognition Assessment Interview (MAI) [23], the Adult Attachment Interview (AAI), the Psychopathy Checklist [24], and the Clinical Diagnostic Interview [25], the CALF is structured, but the interview and interpretation rely primarily on the inference of the underlying processes (contrasts, absence, brevity of explanations, and ability to shift perspectives and to reflect on both emotional, factual and cognitive processes) rather than relying only on the explicit content of the response. The four domains in the LPFS are rated based on the totality of the interview rather than on patient responses to the questions in the corresponding section. For each section, the interviewer has to rate the level of dysfunction by giving a score from 0 to 4, with 0 indicating no impairment and 4 indicating extremely severe impairment.

The CALF opens with questions about general demographics, followed by questions about current problems with mental health and current treatments. The main body of the interview consists of four sections, each of which concerns one of the dimensions in the LPFS, but where some of the questions within each section also provide information on the level of functioning within other domains. Specifically, the CALF prompts patients to talk about the four domains based on their general life situation within the last three to five years.

All sections open with a global question concerning the specific domain, followed by prompts for specific examples and qualifications of the response. All sections conclude with questions about contentment and concerns for the given domain, and whether recent changes, events or periods of higher or lower distress have affected specific areas of functioning within this domain.

Section 1 assesses Self-direction. According to the LPFS, Self-direction concerns the ability to set and pursue realistic and meaningful goals in life. Questions in this section concern the patient’s goals in life, and prompt questions concerning the respondent’s ability to set reasonable goals based on a realistic assessment of personal capacities. Since the pilot testing showed that current life goals may be difficult to evaluate in terms of how realistic they are and how consistently the patient pursues these goals, we included past life goals and how these had been pursued. Following this, the patient is asked about the value and meaning of current and past goals, what has been done to obtain the goals and whether they have been obtained, possible future obstacles, and what the patient can do to overcome these obstacles. Finally, the patient is asked if he or she considers herself to be in control of her life in general, and whether she is satisfied with the goals that he or she is presently pursuing.

Section 2 assesses Intimacy. In the LPFS, Intimacy includes both close relationships and relationships in the community, and is more concerned with the reciprocity and the depth of the relationships than with the size of network of perceived support. In order to identify the degree of reciprocity, this section opens with questions about who the patient sees in daily life and the frequency of contact. Next, the patient is asked to identify a single person who is particularly important and to describe what he or she likes about that person, and then to describe what the other person likes about her. Finally, this section asks about conflicts that have resulted in the discontinuation of social contacts, an area which is also considered in the assessment of the capacity for Empathy.

Section 3 concerns Empathy, which is referred to in the LPFS as the capability to understand and respond adequately to the experiences and motivations of others, and the awareness of how one’s own actions affect others. The section opens with questions about disagreements and who the patient disagrees with. Next, the patient is asked to identify a disagreement with a person, and is asked about the motivations and intentions behind the disagreement (of both those of the patient and the other person), and whether and how the disagreement was resolved. The purpose of these questions is primarily to assess the patient’s capacity for understanding and considering the perspectives and needs of others in a conflict, the ability to understand their reactions, and the ability to learn from disagreements.

Section 4 adds further information concerning the Identity dimension. Usually, responses to the previous sections in the CALF interview are highly salient for the issues covered in the Identity section, because they provide rich information about self-image, self-worth, and the capacity for independent functioning. However, an important aspect of Identity that is not necessarily covered by the previous sections is the patient’s access to and ability to regulate a wide range of emotions. To assess this aspect of Identity, the patient is asked about feelings of sadness, anxiety, anger and pleasure, what triggers these feelings, the intensity and duration of the feeling, and how the patient reacts to the feeling. Finally, the section contains questions about differences between private and public identities.

#### Statistical analysis

The number of patients included was determined primarily on pragmatic grounds. Hence, a post hoc power analysis was conducted to assess the power to assess correlations which indicated that with a sample size of 34, the power to detect a correlation of 0.50 was 86 % with α = 0.05.

For the dependent variable, the LPFS score, we summed the scores on each of the four domains to yield a number that could range from 0 (no dysfunction) to 12 (maximal dysfunction).

To assess the agreement between interviewer ratings and video-based ratings, one of the ratings from experts who had conducted the video rating of the interview was randomly selected, and the rating from that person was correlated with the interviewer’s rating for Pearson correlations. This was done for the individual rating of each domain, as well as for the sum of the four domains. For each correlation, the 95 % confidence intervals were calculated by using Fisher’s Z transformation.

Further, to assess agreement between different video ratings of the same video, we calculated agreement using intraclass correlations from mixed effects regression models. For these correlations, we report the confidence intervals, and the *p*-values based on the assumption that the distribution of the likelihood-ratio test statistic is a 50:50 mixture of *χ*^2^ distributions with k and k + 1° of freedom. All analyses were carried out on Stata 13 for Windows [26].

The analyses were repeated, so that all analyses were first conducted using only the patient sample, and in a second round, the community individuals were included in the analyses.

## Results

In the patient sample, the mean score on the LPFS was 8.18 for the interviewer ratings (range: 1 to 13, standard deviation [SD] = 2.98), and 7.59 for the video ratings (range: 1 to 12, SD = 3.22). In the community sample, the mean score for interviewer rating was 0.86 (range: 0 to 4, SD = 1.46), and the mean video rating was 2.00 (range: 0 to 8, SD = 2.89).

### Agreement between video ratings and interviewer ratings

For five patients, the interviewer did not rate the LPFS, leaving 31 patients for this analysis (17 psychiatric patients and 14 patients with substance use disorders). The power to detect a correlation of 0.5 was 83 % with a sample size of 31, which is still within the acceptable range.

**Fig. 1:**
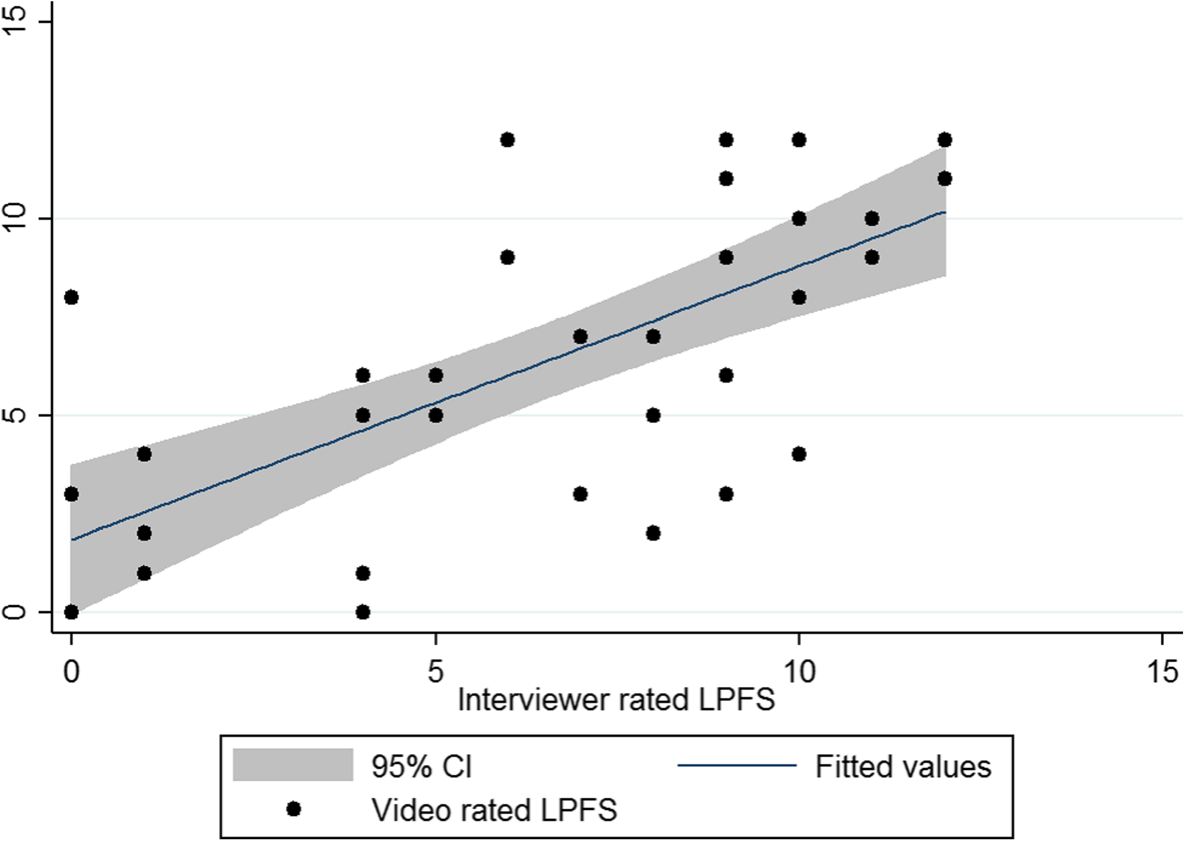
Scatterplot of video-rated LPFS as a function of interviewer-rated LPFS

For the full LPFS, the Pearson correlation between video rating and interviewer was 0.59 (*p* < .001). The domain correlations between the interviewer and video-based ratings are shown in Table 1. Correlations ranged from 0.16 (Identity, ns) to 0.66 (Self-direction, *p* < .001). The correlation between the sum of the LPFS as rated by interviewer and by video rater is illustrated in Fig. 1. When community controls were included, all coefficients increased, and all became significant.

**Table 1:** Pearson correlations between interviewer-rated LPFS and randomly selected video-rated LPFS

	Clinical sample (*n* = 31)	*P*-value	Total sample (*n* = 38)	*P*-value
Identity	.202 (−.164 to .519)	.276	.588 (.331 to .764)	.000
Self-direction	.672 (.417 to .829)	.000	.716 (.514 to .843)	.000
Intimacy	.495 (.171 to .723)	.005	.650 (.417 to .803)	.000
Empathy	.360 (.007 to .634)	.046	.419 (.114 to .651)	.000
Total	.582 (.287 to .776)	.001	.689 (.474 to .827)	.000

Notes: *LPFS* Levels of personality functioning scale

Values in parentheses are 95 % confidence intervals

### Agreement between different video raters

For six patients, only one video had been rated by a video rater, leaving 30 patients for the inter-rater analysis of the video-based ratings. The intraclass correlations between two independent video raters are summarized in Table 2. The intraclass correlations range from 0.31 to 0.60. Again, the highest inter-rater agreement was found for Self-direction, and the weakest for Identity. When community controls were included, all coefficients increased, and all became significant.

**Table 2:** Intraclass correlations between video-rated LPFS

	Clinical sample (*n* = 30)	*P*-value	Total sample (*n* = 37)	*P*-value
Identity	.31 (.09 to .67)	.039	.59 (.38 to .77)	.000
Self-direction	.58 (.35 to .79)	.000	.62 (.41 to .79)	.000
Intimacy	.46 (.22 to .73)	.004	.62 (.42 to .79)	.000
Empathy	.57 (.33 to .78)	.000	.59 (.38 to .78)	.000
Total	.54 (.30 to .77)	.000	.65 (.46 to .81)	.000

Notes: *ICC* Intraclass correlation

*LPFS* Levels of personality functioning scale

Values in parentheses are 95 % confidence intervals

## Discussion

In this study, we tried a semi-structured interview, the CALF, developed to measure the LPFS. The CALF was designed to be conducted in a clinical setting, applying strategies similar to what characterizes assessments in clinical practice, which often involves the inference of the underlying processes in the patient’s narrative. However, the findings were only minimally encouraging for the use of the CALF as a diagnostic instrument for the LPFS. Most inter-rater correlations were statistically significant, but none were in the range where two different assessments were so similar, that one could substitute the other (i.e., more than 50 % shared variance). This is in spite of the fact that we had a very diverse sample of patients in which a wide range of variation could be expected in the LPFS.

In terms of specific domains, the strongest inter-rater reliability was found for Self-direction, both in terms of different video raters and when comparing video ratings to interviewer ratings. Self-direction is characterized by the ability to set and consistently pursue realistic and meaningful goals, and it appears that especially by asking the patient to describe his or her past goals and clarify which of them have been obtained and what has been done to reach them, the interviewer will get a reliable estimate of the patient’s level of functioning in this area. The two interpersonal areas, Empathy and Intimacy, gave more modest inter-rater reliability estimates, and although the correlations were statistically significant and may contribute to the overall clinical picture, such ratings should not be considered stand-alone assessments of these areas of functioning in a research context, let alone in a clinical context. Finally, the inter-rater reliability of the Identity scale was low, indicating that in order to obtain acceptable reliability, a different approach is probably needed than the one found in the current version of CALF. We have no good explanation for the lower reliability on the Identity scale. It may be that Identity is a complicated construct with no universally agreed upon definition e.g. [27], or that the rating of the Identity domain draws more heavily on answers obtained in the other sections of the CALF.

The concerns about the inter-rater reliability in this study are similar to those raised in previous research. Although the present study is a small study that only yields preliminary evidence on the inter-rater reliability of the LPFS, the correlations were strong enough to suggest further research on the rating of the LPFS. A central question is here how a clinical interview format best supports a reliable standardized assessment of the LPFS, and whether using assessment strategies that mirror how psychiatrists and psychologists work in clinical practice may find a place within such format [6]. A major challenge to the dimensional approach in the LPFS is that, unlike specific types of psychopathology, overall personality functioning and the four domains in the LPFS do not manifest in clear, well-defined symptoms, but address complex and diverse phenomena. The question is how this can be reflected in the assessment approach in a way that supports assessment reliability and validity. One extreme would be a fully structured interview in which patients’ answers would be transformed into relevant scores with only slight perceiver inference. The other extreme would be a minimally structured and phenomenologically oriented interview, in which the clinician would infer the LPFS scores based on information which is influenced by several factors besides the patient’s answers [28]. However, although sufficient reliability is easier to obtain when perceiver inference is kept to a minimum, this could come at the price of reduced validity concerning the rating of the complex constructs in the LPFS. Other related studies that have assessed concepts similar to the concepts in the LPFS have used interviews based on a structured format, which also facilitates the patients in talking freely about personal and affect-laden aspects like the CALF, in order to observe metacognitive capacities, narrative coherence, and the representational style of the interviewee, all of which are core elements which influence the assessment of personality functioning. In the Metacognition Assessment Interview (MAI) [23], the patient is asked to describe an autobiographical episode about the worst psychological situation within the last six months, and following this, the clinician adheres to a structured list of questions to assess four functional domains (monitoring, integrating, differentiating and decentering). In the Adult Attachment Interview (AAI), the patient is asked about the demographics of his or her childhood followed with questions about the nature of the relationships with parents, and an elaboration on specific episodes when positive adjectives are used when describing these relationships [29]. Thus, like the CALF, both the MAI and the AAI contain specific questions as well as more open questions about highly personal and affect-laden topics, in which patient statements are used to make inferences about an underlying quality see also [30].

The CALF was designed to assess level of personality functioning in a way that closely mimics what clinicians do in general practice, by requiring the interviewer to prompt for examples and clarifications within broad life functioning areas, rather than by probing for specific behaviors or emotions that match the criteria in the LPFS levels. While it is both a weakness and strength that the CALF also assesses what is left unsaid, this study shows that videotaped assessment interviews can be used to assess the processing of various non-verbal types of data, such as speed of speech, body language and voice intonation [31–33], which would be of particular interest when a patient is being interviewed about salient areas of functioning in life. Given that the present study, like the previous studies that we know of which have assessed the LPFS [20–22], assessed inter-rater agreement based on the rating of same data give a lower bound on the reliability of the assessment. Had different interviews of the same patient been assessed, instead of assessing the same interview, the results would almost certainly have pointed to lower reliability. In turn, this means that correlations with other variables are bound to be considerably lower.

### Directions for further research

The inclusion of underlying processes in patient narratives in the assessment of the LPFS may be highly useful for clinical practice, in which self-reporting may be biased due to impairment in realistic self-appraisal and the ability to reflect upon and understand aspects of functioning, including mental processes and their impact on others. The CALF interview was an attempt to do this in a way that corresponds to how clinicians do in real world settings. However, before assessment of narratives can be meaningfully included in the assessment of personality functioning, improvements in inter-rater reliability are required.

### Limitations

There are a number of important limitations to this study. First, the raters were not a random sample of psychologists and psychiatrists, but rather an expert group with a special interest in PDs. Further, the number of patients and controls was small, and larger samples are needed in future studies. Finally, the interviewers were not blind to the patients’ clinical status.

## Conclusion

The present study showed that only weak inter-rater reliability was obtained, when the Clinical Assessment of the Level of Personality Functioning Scale interview was used to assess the Levels of Personality Functioning Scale. The interview was designed to rely on a relatively high level of inference, and the weak reliability is likely to be an effect of this fact. Based on the present findings, rating based on the Clinical Assessment of the Level of Personality Functioning Scale interview should not be considered a stand-alone assessment of areas of functioning for a given patient.
